# Supplementary material for: Serum and cerebrospinal fluid host proteins indicate stroke in children with tuberculous meningitis
Source: PLoS One. 2021 Apr 30;16(4):e0250944. doi: 10.1371/journal.pone.0250944 (PMC8087017; doi:10.1371/journal.pone.0250944)
Supplement: S1 Table — (PDF) [file pone.0250944.s002.pdf]

S1 Table: List of all host proteins evaluated in serum and cerebrospinal fluid using Luminex multiplex immunoassay and the suppliers of the reagent kits.

**Reagents kits purchased from Merck Millipore, Billerica, MA, USA**	
**Panel names and catalogue numbers**	**Analytes included in the kits**
Human Cardiovascular Disease (CVD) Magnetic Bead Panel 2 (HCVD2MAG-67K)	Von Willebrand factor-cleaving protease (ADAMTS13), D-dimer, growth differentiation factor (GDF)-15, Myoglobin, soluble intracellular adhesion molecule (sICAM)-1, myeloperoxidase (MPO), P-selectin, Neutrophil gelatinase-associated lipocalin (NGAL)/ lipocalin2, soluble vascular adhesion molecule (sVCAM)-1, serum amyloid A (SAA)
Human Neurodegenerative Disease Magnetic Bead Panel 1 (HNDG1MAG-36K)	Apolipoprotein (Apo)-AI, Apo-CIII, complement factor H (CFH), complement Component 3 (CC3)
Human Neurodegenerative Disease Magnetic Bead Panel 2 (HNDG2MAG-36K)	C reactive protein (CRP), alpha-1-Antitrypsin (A1AT), pigment epithelium-derived factor (PEDF), serum amyloid P (SAP), Macrophage inflammatory protein (MIP)-4/ CCL18, complement C4 (CC4)
Human Neurodegenerative Disease Magnetic Bead Panel 3 (HNDG3MAG-36K)	Brain-derived neurotrophic factor (BDNF), cathepsin D, ICAM-1, MPO, platelet derived growth factor (PDGF)-AA, Regulated upon activation, normally T-expressed, and presumably secreted (RANTES)/ CCL5, neural cell adhesion molecule (NCAM), PDGF-AA/BB, VCAM-1, plasminogen activator inhibitor (PAI)- 1 total
Human Neurodegenerative Disease Magnetic Bead Panel 4 (HNDG4MAG-36K)	S100 calcium-binding protein B (S100B), amyloid beta 1-40 (Aβ40), Aβ42, soluble receptor for advanced glycation end products (sRAGE) , glial cell-derived neurotrophic factor (GDNF)
Human Complement Magnetic Bead Panel 1 (HCMP1MAG-19K)	Complement C2 (CC2), CC5, CC4b, CC5a, CC9, complement factor D (CFD), mannose binding lectin (MBL), complement factor 1 (CF1)

**Reagent kits purchased from R&D SYSTEMS Inc. (Biotechne®), Minneapolis, USA**	
Human Magnetic Luminex Screening Assay (LXSAHM- 24)	CCL1 (I-309), CCL4 (macrophage inflammatory protein (MIP)-1β), CXCL8 (IL-8), granulocyte-macrophage colony-stimulating factor (GM-CSF), interleukin (IL)-10, IL-17A, IL-6, matrix metalloproteinase (MMP)-8, CCL2 (monocyte chemoattractant protein (MCP)-1), CD40 ligand (CD40L), Monokine induced by interferon gamma (MIG)/ CXCL9 (MIG), interferon (IFN)-γ, IL-12/23p40, IL-21, IL-7, transforming growth factor (TGF)-α, CCL3/Macrophage inflammatory protein (MIP)-1α, CXCL10/ interferon gamma inducible protein (IP)-10, granulocyte colony-stimulating factor (G-CSF), IL-1β, IL-13, IL-4, MMP-1, tumour necrosis factor (TNF)-α
Human Magnetic Luminex Screening Assay (LXSAHM- 03)	MMP-7, CD56 (NCAM-1), vascular endothelial growth factor (VEGF)-A
Human Magnetic Luminex Screening Assay (LXSAHM- 02)	Ferritin, MMP-9
