# Supplementary material for: Serum and cerebrospinal fluid host proteins indicate stroke in children with tuberculous meningitis
Source: PLoS One. 2021 Apr 30;16(4):e0250944. doi: 10.1371/journal.pone.0250944 (PMC8087017; doi:10.1371/journal.pone.0250944)
Supplement: S2 Table — The least square (LS) means (95% Confidence intervals) of all host markers and accuracies in indicating stroke amongst children with TBM are shown. #reported in ng/ml, all other markers are reported in pg/ml. (PDF) [file pone.0250944.s003.pdf]

S2 Table: Expression of CSF host inflammatory biomarkers amongst study participants with TBM and stroke/no stroke at admission, and utility of individual CSF host protein biomarkers in indicating stroke in TBM patients. The least square (LS) means (95% Confidence intervals) of all host markers and accuracies in the indication of stroke amongst children with TBM are shown. #reported in ng/ml, all other markers are reported in pg/ml.

Marker	TBM-stroke	TBM-no stroke	Not-TBM	P value[a]	P value[b]	P value[c]	Utility in indicating stroke in TBM patients			
							AUC (95% CI)	Cut-off value	Sensitivity %	Specificity %
sVCAM-1	83883.4 (50069.4-117697.3)	161261.8 (119088.4-203435.2)	64955.1 (39129.2-90780.9)	0.0060	0,3748	0,0003	0.79 (0.60-0.98)	120343.5	77.80	71.40
MMP-1	493.5 (288.6-698.5)	942.7 (687.0-1198.3)	462.5 (306.0-619.0)	0.0083	0,8094	0,0024	0.76 (0.53-0.99)	480.355	77.80	71.40
sRAGE	14.8 (12.8-16.9)	10.4 (7.8-12.9)	14.8 (13.2-16.3)	0.0086	0,9603	0,0049	0.78 (0.53-1.00)	13.14	92.90	66.70
CXCL10/IP-10	36270.7 (26910.6-45630.8)	16554.0 (4879.9-28228.2)	7338.6 (189.7-14487.5)	0.0110	0,0000	0,1818	0.73 (0.51-0.95)	7877.2	85.70	66.70
PDGF-AA	11.3 (7.3-15.3)	18.4 (13.4-23.4)	7.1 (4.0-10.1)	0.0297	0,0952	0,0003	0.71 (0.46-0.95)	18.29	55.60	85.70
#LIPOCALIN-2/NGAL	118.7 (88.9-148.4)	38.7 (1.6-75.8)	11.1 (-11.6-33.9)	0.0330	0,0002	0,4799	0.82 (0.63-1.00)	45.88	78.60	77.80
#Apo CIII	115.9 (30.0-201.8)	248.3 (141.2-355.5)	79.1 (12.0-146.1)	0.0584	0,4989	0,0099	0.76 (0.54-0.98)	85.14	88.90	78.60
GM-CSF	92.9 (75.11-110.6)	66.0 (43.8-88.1)	36.4 (22.9-50.0)	0.0627	0,0000	0,0265	0.70 (0.43-0.98)	71.08	85.70	66.70
#D-dimer	91101.8 (69953.6-112250.0)	43555.6 (17179.2-69932.0)	20614.5 (4462.4-36766.7)	0.0645	0,0000	0,4752	0.76 (0.57-0.95)	712.58	100.00	55.60
Ferritin	6863.4 (3512.1-10214.6)	11516.7 (7337.0-15696.4)	3129.6 (570.1-5689.2)	0.0870	0,0812	0,0013	0.66 (0.41-0.91)	6166.45	66.70	71.40
BNDF	0.9 (0.5-1.2)	0.4 (-0.0-0.8)	0.6 (0.3-0.9)	0.0893	0,2117	0,4231	0.75 (0.50-1.00)	0.175	92.90	66.70
GDNF	2.3 (1.8-2.9)	1.1 (0.5-1.8)	1.9 (1.5-2.3)	0.0969	0,2010	0,3372	0.80 (0.57-1.00)	2.11	71.40	77.80
CATHEPSIN	68033.5 (47411.5-88655.5)	94923.7 (69203.5-120643.8)	76489.0 (60738.6-92239.3)	0.1073	0,5148	0,2245	0.65 (0.37-0.93)	88682.78	55.60	92.90
#Adipsin/Complement factor D	77.5 (22.3-132.7)	149.3 (80.4-218.2)	149.3 (36.7-121.1)	0.1081	0,9668	0,0859	0.60 (0.33-0.88)	69.655	55.60	71.40

#ADAMT S13	13.2 (8.3-18.1)	6.8 (0.7-13.0)	7.7 (4.0-11.5)	0.1112	0,0826	0,8049	0.73 (0.47-1.00)	6.525	92.90	66.70
#PEDF	761.2 (654.6-867.7)	898.4 (765.4-1031.3)	714.7 (633.3-796.1)	0.1118	0,4888	0,0220	0.72 (0.50-0.94)	747.445	77.80	71.40
#A1AT	3915.0 (2677.6-5152.4)	2337.9 (794.6-3881.2)	1079.2 (134.1-2024.3)	0.1153	0,0007	0,1680	0.72 (0.49-0.95)	1317.25	85.70	66.70
sICAM1	2680.3 (1368.8-3991.8)	4298.5 (2662.8-5934.2)	1544.8 (543.1-2546.5)	0.1270	0,1725	0,0059	0.71 (0.49-0.94)	2895.005	66.70	78.60
Apo AI	2670.8 (1069.1-4272.5)	4585.3 (2587.6-6582.9)	1431.9 (208.6-2655.2)	0.1390	0,2220	0,0095	0.71 (0.48-0.94)	2038.855	77.80	71.40
CCL3	336.6 (252.8-420.4)	237.3 (132.8-341.8)	185.4 (121.4-249.4)	0.1423	0,0060	0,3980	0.73 (0.49-0.97)	299.535	64.30	77.80
IL-4	229.1 (162.8-295.4)	150.9 (68.2-233.6)	216.1 (165.5-266.8)	0.1442	0,7563	0,1819	0.62 (0.38-0.85)	204.35	42.90	88.90
PAI-1	6130.7 (2972.1-9289.2)	12483.7 (8544.3-16423.0)	2122.6 (-289.8-4535.0)	0.1939	0,0342	0,0283	0.67 (0.39-0.96)	9714.745	66.70	78.60
PDGF-AB/BB	11.2 (7.6-14.8)	18.6 (14.1-23.1)	4.5 (1.7-7.2)	0.2069	0,0245	0,0092	0.74 (0.50-0.98)	13.485	77.80	71.40
#SAP	95.2 (35.0-155.3)	155.5 (80.4-230.5)	62.8 (16.8-108.7)	0.2131	0,3930	0,0394	0.65 (0.40-0.91)	62.555	77.80	64.30
IL-13	991.0 (616.8-1365.3)	625.3 (158.5-1092.1)	545.3 (259.5-831.2)	0.2246	0,2246	0,7696	0.67 (0.43-0.91)	856.895	57.10	77.80
IL-10	48.2 (29.2-67.1)	66.0 (42.3-89.7)	13.9 (-0.90-28.7)	0.2427	0,0063	0,0005	0.63 (0.36-0.89)	49.47	66.70	71.40
TGF-α	17.0 (11.7-22.2)	12.0 (5.5-18.6)	5.6 (1.6-9.6)	0.2436	0,0012	0,0993	0.65 (0.40-0.90)	8.695	85.70	55.60
#P-Selectin	4.3 (1.8-6.8)	0.6 (-2.6-3.7)	0.6 (-1.4-2.5)	0.2515	0,2526	1,0000	0.81 (0.61-1.00)	0.805	92.90	77.80
MPO	59903.4 (42452.5-77354.3)	75660.9 (53895.8-97426.0)	11952.9 (-1375.4-25281.3	0.2611	0,0001	0,0000	0.44 (0.17-0.70)	59743.38	55.60	57.10
VEGF	160.6 (71.4-249.9)	82.5 (-28.9-193.8)	39.5 (-28.7-107.7)	0.2756	0,0352	0,5107	0.58 (0.33-0.83)	34.635	64.30	55.60
#CC9	44.6 (31.1-58.2)	55.1 (38.2-72.0)	30.9 (20.5-41.2)	0.3357	0,1110	0,0177	0.58 (0.29-0.87)	55.97	55.60	85.70
CCL4	477.7 (344.1-611.4)	381.3 (214.6-548.0)	244.6 (142.5-346.7)	0.3680	0,0077	0,1659	0.72 (0.45-0.99)	317.52	78.60	77.80
IL-7	6.1 (3.0-9.3)	4.0 (0.0-8.0)	6.5 (4.1-9.0)	0.3988	0,8471	0,2781	0.59 (0.35-0.83)	5.355	50.00	66.70
NCAM1	108579.9 (76126.1-141033.8)	86967.3 (46490.3-127444.5)	135130.0 (110343.0-159917.1)	0.4057	0,1969	0,0469	0.57 (0.33-0.81)	107522.1	50.00	77.80

#CC4b	509.1 (387.8-630.5)	586.5(435.2-737.8)	257.5 (164.8-350.1)	0.4258	0,0018	0,0005	0.64 (0.40-0.89)	524.93	77.80	57.10
IL-21	68.4 (36.5-100.4)	50.2 (10.3-90.0)	63.4 (39.0-87.8)	0.4749	0,8009	0,5719	0.48 (0.23-0.74)	44.185	57.10	55.60
LL-37	0.05 (0.05-0.06)	0.05 (0.05-0.06)	0.05 (0.05-0.07)	0.4907	0,8454	0,5576	0.61 (0.36-0.86)	0.0495	66.70	57.10
RANTES	27.6 (18.2-37.1)	38.6 (26.9-50.4)	6.3 (-1.0-13.5)	0.4943	0,0047	0,0080	0.63 (0.38-0.89)	35.125	55.60	71.40
CCL2	1237.0 (719.6-1754.3)	957.8 (312.6-1603.1)	1320.7 (925.5-1715.8)	0.4999	0,7967	0,3391	0.42 (0.16-0.68)	943.6	64.30	44.40
CD40L	604.2 (428.7-779.7)	509.7 (290.8-728.6)	329.7 (195.6-463.7)	0.5011	0,0160	0,1645	0.49 (0.23-0.75)	494.365	64.30	55.60
#CRP	147998.5 (89845.9-206151.0)	178988.0 (106459.0-251517.0)	67347.6 (22932.9-111762.4)	0.5052	0,0315	0,0113	0.58 (0.39-0.77)	115529.9	77.80	35.70
#Myoglobin	4.6 (0.3-8.9)	2.3 (-3.1-7.7)	2.1 (-1.2-5.4)	0.5085	0,3676	0,9567	0.75 (0.52-0.99)	0.48	64.30	77.80
#CFH	4392.1 (1282.6-7501.6)	6001.8 (2123.6-9880.0)	1354.6 (-1071.4-3780.6)	0.5172	0,1277	0,0466	0.74 (0.53-0.95)	1182.3	77.80	64.30
MMP-8	12642.6 (8885.6-16399.6)	8871.5 (4185.7-13557.3)	1630.9 (-1238.6-4500.4)	0.5476	0,0020	0,0537	0.60 (0.35-0.84)	13717.38	42.90	77.80
IL-6	586.3 (349.5-823.1)	846.5 (551.2-1141.8)	74.4 (-106.4-255.3)	0.5497	0,0107	0,0109	0.63 (0.39-0.88)	530.68	66.70	64.30
#CF1	570.0 (305.0-834.9)	695.0 (364.6-1025.4)	361.0 (158.6-563.3)	0.5549	0,2131	0,0893	0.56 (0.30-0.81)	316.865	77.80	42.90
#MBL	23.1 (12.5-33.6)	27.5 (14.4-40.7)	8.1 (0.1-16.2)	0.5953	0,0285	0,0149	0.47 (0.19-0.74)	11.75	44.40	64.30
#CC2	2584.6 (1591.5-3577.8)	2996.5 (1757.8-4235.2)	750.8 (-7.8-1509.3)	0.6037	0,0050	0,0032	0.55 (0.28-0.82)	2228.665	66.70	64.30
CXCL8	1111.0 (702.0-1520.0)	1262.5 (752.4-1772.6)	393.1 (80.7-705.5)	0.6428	0,0073	0,0054	0.51 (0.22-0.80)	1179.51	55.60	78.60
IL-12/23p40	415.5 (191.3-639.8)	345.5 (65.8-625.3)	210.9 (939.6-382.2)	0.6958	0,1510	0,4125	0.5 (0.25-0.74)	241.99	57.10	66.70
IL-17A	20.1 (11.3-28.6)	17.3 (6.5-28.1)	7.8 (1.2-14.4)	0.6967	0,0288	0,1367	0.62 (0.34-0.90)	12.045	71.40	66.70
G-CSF	446.5 (251.7-641.3)	506.8 (263.9-749.7)	167.0 (18.3-315.8)	0.6981	0,0264	0,0205	0.58 (0.33-0.83)	435.56	55.60	71.40
#MIP4	68.8 (52.3-85.3)	54.9 (34.3-75.4)	2.1 (-10.5-14.7)	0.6991	0,0006	0,0030	0.55 (0.30-0.81)	109.15	35.70	100.00
GDF-15	0.4 (0.2-0.7)	0.4 (0.0-0.7)	0.3 (0.1-0.4)	0.7034	0,2764	0,5993	0.69 (0.41-0.98)	0.28	78.60	66.70
#SAA	2282.1 (1317.2-3246.9)	1531.1 (327.7-2734.5)	1531.1 (-216.3-1257.5)	0.7182	0,0314	0,4536	0.73 (0.47-0.99)	5.92	92.90	66.70

CXCL9	14266.0 (10933.1-17598.9)	11260.7 (7103.9-15417.6)	1834.5 (-711.1-4380.0)	0.7218	0,0003	0,0394	0.62 (0.36-0.88)	9538.38	71.40	55.60
IL-1b	46.5 (29.4-63.7)	49.9 (28.5-71.2)	14.6 (1.5-27.7)	0.8075	0,0047	0,0069	0.53 (0.27-0.78)	46.085	66.70	57.10
#CC4	1586.8 (1017.5-2156.2)	1695.5 (985.4-2405.6)	583.9 (149.1-1018.7)	0.8110	0,0071	0,0100	0.60 (0.35-0.85)	1304.765	66.70	64.30
MMP-9	4624.1 (3104.2-6144.0)	4334.3 (2438.8-6229.9)	790.8175 (-370.0-1951.6)	0.8112	0,0002	0,0025	0.57 (0.30-0.84)	4344.495	50.00	66.70
#CC3	1344.3 (676.8-2011.9)	1462.0 (629.5-2294.6)	697.3 (176.5-1218.1)	0.8251	0,1306	0,1236	0.57 (0.32-0.83)	861.065	77.80	57.10
CCL1	210.8 (164.5-257.1)	184.5 (126.8-242.3)	15.8 (-19.6-51.1)	0.8814	0,0000	0,0000	0.60 (0.33-0.87)	153.05	64.30	66.70
CC5a	83.1 (49.7-116.4)	79.4 (37.8-121.0)	37.1 (11.6-62.5)	0.8894	0,0325	0,0876	0.53 (0.26-0.79)	68.4	64.30	66.70
MMP7	190.8 (47.8-333.8)	177.8 (-0.5-356.1)	144.1 (34.9-253.3)	0.9092	0,6034	0,7469	0.76 (0.52-1.00)	91.57	92.90	55.60
Ab42	201.6 (59.0-344.3)	178.8 (0.9-356.7)	354.5 (245.6-463.5)	0.9374	0,1423	0,1287	0.55 (0.29-0.81)	120.305	71.40	55.60
#CC5	576.3 (303.8-848.9)	590.7 (250.8-930.7)	186.4 (-21.8-394.6)	0.9473	0,0268	0,0469	0.61 (0.36-0.87)	465.65	66.70	71.40
IFN-γ	529.2 (396.9-661.6)	495.3 (330.3-660.4)	29.8 (-71.2-130.9)	0.9743	0,0002	0,0162	0.55 (0.28-0.81)	445.145	57.10	55.60
TNF-α	91.1 (62.8-119.4)	86.9 (51.6-122.1)	14.2 (-7.4-35.9)	0.9877	0,0006	0,0434	0.58 (0.30-0.86)	56.05	78.60	55.60
Ab40	656.6 (206.0-1107.2)	648.0 (86.0-1210.0)	1223.5 (879.4-1567.7)	0.9989	0,0765	0,0892	0.51 (0.25-0.76)	569.695	57.10	55.60

[a]Comparison between TBM with stroke and TBM without stroke. [b]Comparison between TBM with stroke and not-TBM. [c]Comparison between TBM without stroke and not-TBM.
