# Supplementary material for: Serum and cerebrospinal fluid host proteins indicate stroke in children with tuberculous meningitis
Source: PLoS One. 2021 Apr 30;16(4):e0250944. doi: 10.1371/journal.pone.0250944 (PMC8087017; doi:10.1371/journal.pone.0250944)
Supplement: S3 Table — The least square (LS) means (95% Confidence intervals) of all host markers and accuracies in indicating of stroke amongst children with TBM are shown. #reported in ng/ml, all other markers are reported in pg/ml. (PDF) [file pone.0250944.s004.pdf]

S3 Table: Expression of serum host protein biomarkers amongst study participants with TBM and stroke/no stroke at admission, and utility of individual serum host protein biomarkers in indicating stroke in TBM patients. The least square (LS) means (95% Confidence intervals) of all host markers and accuracies in the indication of stroke amongst children with TBM are shown. #reported in ng/ml, all other markers are reported in pg/ml.

Marker	TBM-stroke	TBM-no stroke	Not-TBM	P value[a]	P value[b]	P value[c]	Utility in indicating stroke in TBM patients			
							AUC (95% CI)	Cut-off value	Sensitivity %	Specificity %
#D-dimer	4.1 (3.4-4.8)	2.1 (1.2-2.9)	3.6 (3.1-4.1)	0.0187	0,2022	0,0860	0.83 (0.64-1.00)	3.410048	100.00	66.70
IL-13	0.6 (-0.1-1.3)	2.0 (1.1-2.9)	1.3 (0.7-1.9)	0.0194	0,1402	0,1808	0.73 (0.52-0.94)	1.088349	77.80	78.60
#ADAMTS13	3.0 (2.7-3.3)	2.1 (1.7-2.5)	2.7 (2.5-2.9)	0.0314	0,1053	0,1524	0.82 (0.63-1.00)	2.909587	85.70	66.70
CCL2	2.5 (2.3-2.7)	2.2 (2.0-2.4)	2.8 (2.6-2.9)	0.0322	0,0094	0,0000	0.82 (0.63-1.00)	2.311411	92.90	66.70
#SAA	4.9 (3.9-5.8)	2.1 (0.9-3.3)	3.9 (3.2-4.7)	0.0325	0,0797	0,1829	0.75 (0.50-1.00)	3.356864	100.00	66.70
Ferritin	4.9 (3.9-5.8)	2.2 (1.0-3.4)	4.1 (3.3-4.8)	0.0327	0,1303	0,1529	0.77 (0.53-1.00)	4.220998	92.90	66.70
GDF-15	0.4 (0.2-0.6)	0.1 (-0.1-0.3)	0.4 (0.3-0.6)	0.0364	0,9423	0,0196	0.90 (0.78-1.00)	0.296527	78.60	88.90
GNDF	2.2 (1.8-2.6)	1.1 (0.6-1.6)	1.8 (1.5-2.1)	0.0518	0,0974	0,2598	0.72 (0.46-0.97)	1.771286	100.00	55.60
IL-7	1.7 (1.5-1.8)	1.4 (1.1-1.6)	1.4 (1.2-1.5)	0.0549	0,0107	0,8916	0.79 (0.59-1.00)	1.467458	85.70	66.70
MMP-9	5.4 (4.4-6.5)	2.6 (1.2-3.9)	4.4 (3.6-5.2)	0.0560	0,0632	0,2652	0.68 (0.39-0.98)	5.137117	85.70	66.70
CC5a	3.4 (3.3-3.5)	3.6 (3.4-3.7)	3.4 (3.3-3.5)	0.0624	0,9755	0,0450	0.74 (0.51-0.96)	3.414416	88.90	71.40
#LIPOCALIN-2/NGAL	2.7 (2.2-3.3)	1.3 (0.6-2.0)	2.2 (1.8-2.7)	0.0629	0,1071	0,2724	0.74 (0.47-1.00)	2.562729	71.40	77.80
IL-4	1.8 (1.5-2.1)	1.0 (0.6-1.4)	2.1 (1.9-2.3)	0.0673	0,1780	0,0116	0.81 (0.58-1.00)	1.543709	92.90	77.80
#P-Selectin	2.4 (1.9-2.9)	1.2 (0.5-1.8)	1.8 (1.4-2.2)	0.0704	0,0380	0,4370	0.69 (0.41-0.96)	1.006566	100.00	55.60
#Myoglobin	1.2 (0.8-1.5)	0.7 (0.3-1.1)	1.2 (1.0-1.5)	0.0856	0,8942	0,0476	0.74 (0.47-1.00)	0.791936	100.00	66.70
#CC2	4.1 (3.9-4.3)	4.4 (4.1-4.7)	4.3 (4.1-4.5)	0.1027	0,1919	0,4992	0.73 (0.52-0.94)	4.133227	88.90	64.30
MMP-8	4.6 (3.8-5.3)	3.6 (2.7-4.5)	3.8 (3.3-4.4)	0.1086	0,1106	0,6990	0.66 90.37-0.95)	4.074843	100.00	55.60
IL-1b	0.0 (-0.3-0.3)	0.6 (0.2-0.9)	0.5 (0.1-0.6)	0.1154	0,0164	0,7429	0.72 (0.55-0.89)	0.387258	44.40	100.00
#CC4	4.7 (4.5-4.8)	4.8 (4.7-5.0)	4.6 (4.5-4.7)	0.1197	0,4371	0,0202	0.70 (0.45-0.94)	4.608858	77.80	64.30
sRAGE	2.9 (2.9-3.0)	2.8 (2.7-2.9)	2.9 (2.8-3.0)	0.1257	0,5798	0,2724	0.65 (0.37-0.92)	2.910854	78.60	55.60

IL-10	0.1 (-0.2-0.4)	0.5 (0.1-0.9)	0.8 (0.6-1.1)	0.1546	0,0015	0,3626	0.75 (0.55-0.95)	0.177054	66.70	78.60
RANTES	4.9 (4.7-5.1)	5.2 (4.9-5.4)	4.9 (4.8-5.1)	0.1581	0,7191	0,2149	0.73 (0.49-0.97)	5.021645	77.80	64.30
#CC4b	4.4 (4.3-4.5)	4.6 (4.4-4.7)	4.3 (4.2-4.4)	0.1736	0,2584	0,0163	0.79 (0.59-0.98)	4.492727	77.80	71.40
CCL1	1.4 (1.1-1.6)	1.1 (0.8-1.4)	1.3 (1.1-1.4)	0.1819	0,4661	0,3999	0.75 (0.49-1.00)	1.187097	71.40	77.80
#MBL	3.9 (3.5-4.2)	4.2 (3.8-4.7)	3.6 (3.4-3.9)	0.1900	0,2575	0,0187	0.67 (0.42-0.92)	4.30793	55.60	85.70
#SAP	5.7 (5.4-5.9)	5.4 (5.2-5.7)	5.3 (5.1-5.4)	0.2394	0,0082	0,2874	0.71 (0.46-0.95)	5.461748	78.60	66.70
#CC5	4.7 (4.6-4.8)	4.7 (4.5-4.8)	4.6 (4.5-4.6)	0.3149	0,0049	0,1583	0.45 (0.15-0.75)	4.739838	71.40	55.60
MMP7	2.9 (2.4-3.5)	2.1 (1.4-2.8)	2.8 (2.4-3.3)	0.3231	0,8378	0,4824	0.55 (0.22-0.88)	2.73654	92.90	55.60
IFN-γ	0.3 (-0.2-0.8)	0.7 (0.1-1.3)	0.5 (0.1-0.8)	0.3237	0,5436	0,5754	0.59 (0.40-0.78)	0.71536	33.30	85.70
Apo AI	5.5 (5.4-5.5)	5.5 (5.5-5.6)	5.4 (5.3-5.5)	0.3302	0,2416	0,0417	0.67 (0.45-0.90)	5.465526	77.80	57.10
IL-17A	0.0 (-0.3-0.3)	0.3 (-0.1-0.7)	0.6 (0.3-0.8)	0.3508	0,0045	0,5622	0.61 (0.47-0.76)	0.544245	22.20	100.00
#Adipsin/Complement factor D	3.3 (3.2-3.4)	3.5 (3.3-3.6)	3.5 (3.4-3.6)	0.3522	0,0010	0,9611	0.44 (0.17-0.71)	3.266696	55.60	64.30
MPO	6.5 (6.3-6.6)	6.6 (6.4-6.8)	6.5 (6.4-6.6)	0.3680	0,9841	0,3339	0.6 (0.35-0.84)	6.684858	55.60	64.30
#CC9	3.5 (3.4-3.6)	3.6 (3.4-3.7)	3.5 (3.5-3.6)	0.3706	0,4302	0,7627	0.45 (0.18-0.73)	3.511713	55.60	64.30
PDGF-AB/BB	4.6 (4.4-4.9)	4.8 (4.5-5.1)	4.4 (4.3-4.6)	0.3949	0,1346	0,0295	0.70 (0.45-0.95)	4.746888	77.80	78.60
G-CSF	1.2 (0.7-1.8)	0.9 (0.2-1.6)	1.9 (1.5-2.3)	0.4012	0,0669	0,0146	0.57 (0.31-0.83)	1.092559	64.30	66.70
#PEDF	4.3 (4.3-4.4)	4.4 (4.3-4.5)	4.3 (4.3-4.4)	0.4414	0,8270	0,3048	0.58 (0.29-0.87)	4.347646	55.60	64.30
LL-37	0.2 (0.1-0.2)	0.2 (0.1-0.3)	0.2 (0.1-0.2)	0.4455	0,8111	0,5324	0.56 (0.29-0.82)	0.162206	66.70	57.10
CCL3	1.2 (0.6-1.7)	1.5 (0.8-2.3)	1.4 (1.0-1.9)	0.4513	0,4706	0,8388	0.70 (0.46-0.94)	1.697793	66.70	85.70
#CFH	5.6 (5.5-5.7)	5.6 (5.5-5.7)	5.5 (5.4-5.6)	0.4803	0,0376	0,0119	0.54 (0.27-0.81)	5.624647	55.60	57.10
CD40L	4.1 (4.0-4.2)	4.0 (3.9-4.2)	4.0 (3.9-4.1)	0.4904	0,3567	0,9674	0.61 (0.35-0.87)	4.054332	71.40	55.60
NCAM	5.6 (5.5-5.7)	5.5 (5.4-5.7)	5.7 (5.6-5.7)	0.5197	0,3882	0,1516	0.52 (0.26-0.77)	5.641184	50.00	66.70
#MIP4	2.4 (2.3-2.6)	2.4 (2.2-2.5)	2.3 (2.2-2.4)	0.5491	0,1611	0,5739	0.58 (0.32-0.83)	2.446304	50.00	77.80
BNDF	4.1 (3.8-4.4)	4.2 (3.9-4.5)	4.1 (3.9-4.3)	0.5639	0,9763	0,5451	0.67 (0.41-0.94)	4.180373	77.80	64.30
#Apolipoprotein CIII	5.1 (5.0-5.3)	5.2 (5.0-5.4)	5.0 (4.9-5.1)	0.5835	0,0862	0,0402	0.43 (0.18-0.68)	5.177141	55.60	57.10
IL-6	0.7 (0.4-1.1)	0.9 (0.5-1.3)	1.1 (0.9-1.4)	0.5899	0,0705	0,3221	0.60 (0.35-0.84)	1.172747	44.40	78.60
sICAM1	5.3 (5.2-5.4)	5.3 (5.2-5.5)	5.4 (5.3-5.5)	0.6260	0,2193	0,5949	0.59 (0.34-0.83)	5.371051	55.60	71.40
CATHEPSIN-D	5.6 (5.5-5.8)	5.7 (5.5-5.9)	5.7 (5.6-5.9)	0.6418	0,2993	0,6971	0.53 (0.26-0.80)	5.817493	44.40	78.60

TNF-a	0.6 (0.3-0.9)	0.7 (0.3-1.1)	1.2 (0.9-1.5)	0.7378	0,0074	0,0467	0.53 (0.28-0.78)	1.011099	44.40	71.40
PDGF-AA	3.9 (3.7-4.1)	4.0 (3.7-4.3)	3.7 (3.6-3.9)	0.7450	0,2877	0,2060	0.56 (0.30-0.82)	3.931484	66.70	64.30
Ab42	0.8 (0.1-1.5)	0.6 (-0.2-1.5)	1.2 (0.6-1.7)	0.7622	0,4039	0,2959	0.53 (0.34-0.72)	1.373263	28.60	77.80
PAI-1	5.5 (5.4-5.7)	5.5 (5.3-5.7)	5.4 (5.3-5.5)	0.7890	0,0346	0,1208	0.48 (0.21-0.76)	5.444422	64.30	44.40
CXCL8	1.5 (1.3-1.8)	1.5 (1.2-1.8)	1.7 (1.5-1.9)	0.7921	0,2157	0,1775	0.57 (0.31-0.83)	1.547338	57.10	55.60
CC4	5.1 (4.5-5.7)	5.2 (4.5-6.0)	4.5 (4.0-4.9)	0.7955	0,1042	0,0939	0.65 (0.40-0.90)	5.295138	55.60	85.70
IL-21	0.2 (-0.1-0.6)	0.2 (-0.3-0.6)	0.5 (0.2-0.8)	0.8134	0,3436	0,2846	0.48 (0.31-0.65)	1.174291	14.30	100.00
TGF-a	1.7 (1.3-2.0)	1.6 (1.2-2.1)	1.3 (1.0-1.6)	0.8377	0,0872	0,2074	0.59 (0.32-0.87)	1.62864	71.40	66.70
CXCL10	1.9 (1.5-2.2)	1.9 (1.5-2.3)	2.2 (1.9-2.4)	0.8452	0,1440	0,2927	0.54 (0.26-0.82)	1.631567	55.60	85.70
#CRP	5.1 (4.9-5.4)	5.2 (4.8-5.5)	5.0 (4.8-5.3)	0.8463	0,5412	0,4615	0.57 (0.39-0.75)	5.361477	88.90	28.60
CXCL9	2.5 (1.6-3.4)	2.6 (1.5-3.8)	2.2 (1.5-2.9)	0.8599	0,5741	0,4993	0.5 (0.25-0.75)	3.458014	44.40	71.40
sVCAM-1	6.0 (5.9-6.2)	6.1 (5.9-6.2)	6.2 (6.2-6.4)	0.8801	0,0009	0,0060	0.60 (0.34-0.86)	6.081211	66.70	64.30
VEGF	2.2 (2.1-2.4)	2.2 (2.0-2.4)	2.1 (2.0-2.2)	0.9155	0,1797	0,2965	0.50 (0.25-0.75)	2.211969	64.30	55.60
#A1AT	4.4 (4.2-4.5)	4.4 (4.2-4.6)	4.2 (4.1-4.4)	0.9201	0,1951	0,2207	0.57 (0.30-0.84)	4.265778	55.60	64.30
MMP-1	3.7 (3.5-3.9)	3.7 (3.5-4.00	3.5 (3.3-3.6)	0.9262	0,0695	0,0957	0.56 (0.30-0.81)	3.638005	66.70	50.00
#CF1	4.8 (4.7-5.0)	4.8 (4.6-5.0)	4.8 (4.6-4.9)	0.9263	0,4473	0,5796	0.48 (0.24-0.73)	4.780595	50.00	66.70
CCL4	2.1 (1.8-2.4)	2.1 (1.7-2.5)	2.6 (2.3-2.9)	0.9415	0,0226	0,0572	0.61 (0.35-0.88)	2.234462	55.60	85.70

[a]Comparison between TBM with stroke and TBM without stroke. [b]Comparison between TBM with stroke and not-TBM. [c]Comparison between TBM without stroke and not-TBM.
